# Comparison of Clinical and Laboratory Characteristics in Lupus Nephritis vs. Non-Lupus Nephritis Patients—A Comprehensive Retrospective Analysis Based on 921 Patients

**DOI:** 10.3390/jcm13154486

**Published:** 2024-07-31

**Authors:** Joanna Kosałka-Węgiel, Radosław Dziedzic, Andżelika Siwiec-Koźlik, Magdalena Spałkowska, Mamert Milewski, Anita Wach, Lech Zaręba, Stanisława Bazan-Socha, Mariusz Korkosz

**Affiliations:** 1 Jagiellonian University Medical College, Department of Rheumatology and Immunology, Jakubowskiego 2, 30-688 Kraków, Poland; mariusz.korkosz@uj.edu.pl; 2 University Hospital, Department of Rheumatology, Immunology and Internal Medicine, Jakubowskiego 2, 30-688 Kraków, Poland; lek.andzelika.siwiec@gmail.com (A.S.-K.); mamert.mmm@gmail.com (M.M.); anita.wach@gmail.com (A.W.); stanislawa.bazan-socha@uj.edu.pl (S.B.-S.); 3 Jagiellonian University Medical College, Doctoral School of Medical and Health Sciences, Św. Łazarza 16, 31-530 Kraków, Poland; radoslawjozefdziedzic@gmail.com; 4 Jagiellonian University Medical College, Department of Dermatology, Botaniczna 3, 31-501 Kraków, Poland; magdalena.spalkowska@uj.edu.pl; 5 University of Rzeszów, College of Natural Sciences, Institute of Computer Science, Pigonia 1, 35-310 Rzeszów, Poland; lzareba@ur.edu.pl; 6 Jagiellonian University Medical College, Department of Internal Medicine, Faculty of Medicine, Jakubowskiego 2, 30-688 Kraków, Poland

**Keywords:** systemic lupus erythematosus, lupus nephritis, prognostic factors, EULAR/ACR, ISN/RPS

## Abstract

**Background**: Lupus nephritis (LN) is an inflammation of the kidneys that is related to systemic lupus erythematosus (SLE). This study aimed to evaluate the differences in clinical and laboratory characteristics between LN and non-LN SLE patients. **Methods**: We conducted a retrospective analysis of medical records collected from SLE patients treated at the University Hospital in Kraków, Poland, from 2012 to 2022. All patients met the 2019 European League Against Rheumatism and the American College of Rheumatology (EULAR/ACR) criteria for SLE. **Results**: Among 921 SLE patients, LN was documented in 331 (35.94%). LN patients were younger at SLE diagnosis (29 vs. 37 years; *p* < 0.001) and had a male proportion that was 2.09 times higher than the non-LN group (16.62% vs. 7.97%; *p* < 0.001). They were more often diagnosed with serositis and hematological or neurological involvement (*p* < 0.001 for all). Hypertension and hypercholesterolemia occurred more frequently in these patients (*p* < 0.001 for both). LN patients exhibited a higher frequency of anti-dsDNA, anti-histone, and anti-nucleosome antibodies (*p* < 0.001 for all). Conversely, the non-LN group had a 1.24-fold (95% CI: 1.03–1.50; *p* = 0.021) increase in the odds ratio of having positive anti-cardiolipin IgM antibody results. LN patients were more frequently treated with immunosuppressants. The risk factors for experiencing at least three LN flares included female sex, younger age at the onset of LN or SLE, LN occurring later than SLE onset, the presence of anti-nucleosome or anti-dsDNA antibodies, and certain SLE manifestations such as myalgia, arthritis, proteinuria > 3.5 g/day, and pathological urinary casts in the urine sediment. **Conclusions**: LN patients differ from non-LN patients in the age of SLE diagnosis, treatment modalities, and autoantibody profile and have more frequent, severe manifestations of SLE. However, we still need more prospective studies to understand the diversity of LN and its progression in SLE patients.

## 1. Introduction

Systemic lupus erythematosus (SLE) is an autoimmune disease characterized by the abnormal activation of autoreactive T and B cells, subsequent production of autoantibodies, activation of complement, and immune-complex deposition, which results in tissue and also organ damage [1,2,3]. SLE is diagnosed predominantly in women of young age, interestingly, with a female-to-male ratio of about 15:1 [4]. Other risk factors for SLE development include race other than Caucasian, genetic determinants (i.e., gene variants located on the X chromosome, such as *IRAK1*, *MECP2*, and *TLR7*), hormonal factors (i.e., estrogens, progesterone, and prolactin), immune abnormalities, and environmental factors (i.e., ultraviolet light exposure, urban areas, cigarette smoking, and viral and bacterial infections) [5,6,7,8].

Kidney involvement is one of the most common and severe manifestations of SLE, affecting up to 75% of patients during the course of the disease [9,10,11]. It typically develops in the early stages of SLE, especially within the first 3–5 years, but it can also present at initial diagnosis [12]. The manifestation of lupus nephritis (LN) varies from subclinical laboratory abnormalities to overt nephritis, nephrotic syndrome, and rapidly progressive renal failure [13,14]. Additionally, up to 30% of patients with LN will develop ESKD within 5 years of onset [12]. Risk factors for progressive kidney disease are not fully recognized but include neuropsychiatric lupus, pediatric onset, male sex, race other than Caucasian, poor socioeconomic status, hypertension, impaired renal function at the time of renal biopsy, anemia, presence of anti-dsDNA antibodies, persistent hypocomplementemia, frequent relapses or incomplete remission, and proteinuria > 4 g per day at diagnosis [10,15,16].

Histologically, there are six distinct classes of nephropathy classified by the International Society of Nephrology/Renal Pathology Society (ISN/RPS) that represent different manifestations and severities of renal involvement in SLE [9]. Patients with proliferative forms of LN are at the highest risk for kidney replacement therapy [12]. Additionally, crescentic glomerulonephritis, thrombotic microangiopathy, or extensive tubulointerstitial damage increase the risk for a worse renal prognosis in LN patients [15,16]. Patients with LN have a higher mortality ratio and die earlier than SLE patients without LN [12]; therefore, early LN diagnosis and prompt treatment initiation are vital to prevent disease progression. Many studies have been carried out on LN cases to determine the predictors of a more unfavorable prognosis; however, their results are inconsistent [11,17,18]. Furthermore, data on the Polish LN population remains scarce [19,20,21,22]. Thus, we aimed to retrospectively evaluate the clinical and laboratory data, including histology, disease follow-up, and treatment modalities, in a large cohort of 921 Polish SLE patients, including 331 subjects with LN. We also examined which factors impact LN development and further prognosis, which could be useful for clinicians.

## 2. Patients and Methods

### 2.1. Study Population

We retrospectively reviewed the medical records of all SLE cases diagnosed and treated in the University Hospital, Kraków, Poland, from January 2012 to June 2022. At the time of data collection, all patients met the European League Against Rheumatism and the American College of Rheumatology (EULAR/ACR) criteria from 2019 for SLE [23].

This paper is a continuation of our previous manuscript on LN, in which detailed information on our methods has been provided [24]. Briefly, we recorded data on sex, current age, age at first SLE symptoms and diagnosis, the time between the onset of SLE symptoms and diagnosis, duration of the disease, family history of SLE and other autoimmune diseases, clinical and laboratory SLE manifestations, internist comorbidities, miscarriages in women, different treatment modalities, and cause of and age at death (if applicable). The evaluated clinical manifestations included general symptoms, lymphadenopathy, skin lesions, oral or nasopharyngeal ulcerations, photosensitivity, joint involvement, serositis, hematologic domain (leukopenia, lymphopenia, anemia, hemolytic anemia, thrombocytopenia, macrophage activation syndrome, and thrombotic thrombocytopenic purpura), kidney, nervous system and respiratory tract involvement, Raynaud’s phenomenon, and lupoid hepatitis. All of them were defined in detail in our previous paper [24]. We also collected data on family history concerning SLE and other autoimmune diseases in the first- and second-line degrees of the ascending and descending relatives.

Next, we divided patients into two subgroups: the first comprised those with LN diagnosis (LN patients), and the second consisted of patients without LN diagnosis (non-LN patients). LN was confirmed either by a renal biopsy and classified according to the ISN/RPS criteria or based on overt renal symptoms (proteinuria, active urinary sediment) during a lupus flare [2]. The evaluation of LN was extended to age at LN diagnosis, histologic type of nephropathy according to the ISN/RPS criteria (if kidney biopsy was performed), numbers of LN exacerbations, and diagnosis of ESKD, if applicable [25]. We also analyzed internal disease comorbidities such as arterial hypertension, diabetes mellitus, hypercholesterolemia, atrial fibrillation, lower extremity peripheral artery disease, heart failure, malignant tumor, or any thromboembolic events. The recorded treatment modalities included corticosteroids, hydroxychloroquine or chloroquine, azathioprine, methotrexate, cyclosporine A, mycophenolate mofetil, cyclophosphamide, sulfasalazine, immunoglobulins intravenously in suppressive doses, and biological agents (belimumab, rituximab, and anifrolumab) used currently or in the past. We also reported if a patient had a splenectomy or plasmapheresis in their medical records.

We received approval for the research from the Bioethics Committee of the Jagiellonian University Medical College (No: 118.6120.41.2023, on 15 June 2023). Furthermore, all procedures adhered to the ethical principles outlined in the Declaration of Helsinki.

### 2.2. Laboratory Analysis

We used routine laboratory techniques to measure complete blood cell count (CBC), lipid profile, haptoglobin, creatinine with estimated glomerular filtration rate (eGFR, using Modification of Diet in Renal Disease formula), 24 h urine protein excretion, urinary sediment analysis, direct antiglobulin test, and blood group designation [26]. Anti-nuclear antibodies (ANAs) were evaluated by an indirect immunofluorescence (IIF) technique using Hep-2 cells. Extractable Nuclear Antigen (ENA) testing was conducted when ANA (IIF) results were positive. Anti-Sjögren’s-syndrome-related antigen A (SSA), anti-Sjögren’s-syndrome-related antigen B (SSB), anti-histone, anti-nucleosome, anti-Smith (Sm), and anti-ribonucleoprotein (RNP) antibodies and autoantibodies were identified by an enzyme-linked immunosorbent assay (ELISA) or a line-blot immunoassay. Anti-double-stranded DNA (anti-dsDNA) antibodies were assayed by IIF using *Crithidia luciliae* as a substrate. Anti-myeloperoxidase (MPO) and anti-proteinase three (PR3) antibodies were assessed using a standardized ELISA technique. Serum complement levels (C3c and C4) and rheumatoid factor (RF) were assessed by nephelometry. Laboratory tests for hypercoagulability were also included, such as lupus anticoagulant (LA), anti-cardiolipin (aCL), anti-beta-2-glycoprotein I (anti-β2GPI) antibodies (both in IgM and IgG classes), antithrombin activity, protein C activity, free protein S level, activity of factor VIII, and presence of factor V Leiden and prothrombin G20210A gene variants. All of them were measured using routine laboratory techniques.

### 2.3. Statistical Elaboration

The results were analyzed using STATISTICA Tibco 13.3 software (StatSoft Inc., Tulsa, OK, USA). Categorical variables are presented as frequencies (number of cases) with relative frequencies (percentages) and compared using the Chi^2^ test or the exact Fisher test. The normality of data distribution was evaluated using the Shapiro–Wilk test. All continuous variables were non-normally distributed and thus were presented as median with Q1–Q3 ranges and compared using the Mann–Whitney test. To calculate the odds ratio (OR) with a 95% confidence interval (CI), the cut-off points were calculated based on receiver operating characteristic (ROC) curves. Cluster analysis was performed using the k-means method. A significance threshold of two-sided *p*-values below 0.05 was employed for all analyses.

## 3. Results

### 3.1. Demographic Characteristics

The summary of demographic parameters is provided in Table 1. The study included 921 SLE patients. Among them, 331 (35.94%) represented the LN cases, with the most common being class IV (diffuse proliferative glomerulonephritis), identified in 91 (50.56%) out of 180 performed renal biopsies. Detailed characteristics of the kidney specimen histology are provided in our previous publication [24].

The treatment modalities based on the kidney biopsy classes are summarized in Table 2. As presented, LN patients regarding the class of nephropathy differ in the frequency of usage of specific immunosuppressive medications such as cyclophosphamide and plasmapheresis.

In 207 (62.53%) LN patients, kidney manifestations were present at SLE diagnosis, while in 122 (36.86%) patients, it was diagnosed at a median of 5.5 years later (*p* < 0.001). Patients with confirmed LN were diagnosed with SLE at a median of 8 years earlier (29 vs. 37 years, respectively; *p* < 0.001) and two-fold more frequently in childhood or teenage years (*p* < 0.001), with the first symptoms appearing at a median of 6 years earlier (28 vs. 34 years, respectively; *p* < 0.001) than in the remaining group. Consequently, the time delay between symptom onset and diagnosis was a median of 0.5 years shorter in the LN group (0 vs. 0.5 years, respectively; *p* < 0.001). On the other hand, the disease duration from diagnosis to analysis was similar in both groups (median: 13 vs. 14 years, respectively; *p* = 0.76). Women constituted the majority of cases in both SLE subgroups; however, SLE was diagnosed 2.09 times more frequently in men in the LN cases (*p* < 0.001).

In 157 individuals (17.05%), there were reported cases of systemic autoimmune disorders among close relatives, with no significant differences observed between the two studied subgroups (*p* > 0.05). Additionally, Hashimoto’s disease was reported in 16 individuals (1.74% overall), type 1 diabetes mellitus in 4 individuals (0.43% overall), Graves–Basedov disease in 3 individuals (0.33% overall), Sjögren’s syndrome in 2 individuals (0.22% overall), systemic sclerosis in 1 individual (0.11% overall), granulomatosis with polyangiitis in 1 individual (0.11% overall), dermatomyositis in 1 individual (0.11% overall), mixed connective tissue disease in 1 individual (0.11% overall), celiac disease in 1 individual (0.11% overall), ulcerative colitis in 1 individual (0.11% overall), myasthenia gravis in 1 individual (0.11% overall), immune thrombocytopenia in 1 individual (0.11% overall), autoimmune hepatitis in 1 individual (0.11% overall), Addison–Biermer anemia in 1 individual (0.11% overall), and undifferentiated connective tissue disease in 1 individual (0.11% overall).

### 3.2. Lupus Nephritis Is Related to More Severe Clinical Immunosuppressive Treatment

Table 3 presents the frequencies of systemic involvement other than kidney-related involvement in the SLE cohort. In the LN group, the most common were hematological (95.17%), joint (84.29%), and constitutional symptoms (80.97%). Non-LN patients exhibited similar predominant clinics, with joint (92.88%), hematological (89.66%), and mucocutaneous signs (87.3%) being the most frequent. Comparing LN to non-LN cases, the former group was characterized by more severe manifestations. For instance, diffuse alveolar hemorrhage (4.75 times; *p* = 0.024), central nervous system involvement (3.03 times; *p* < 0.001), pleural effusion (2.22 times; *p* < 0.001), and pericardial effusion (2.22 times; *p* < 0.001) were more common in LN. Additionally, we reported fever (1.33 times; *p* < 0.001), fatigue or weakness (1.11 times; *p* = 0.037), hematological signs (1.06 times; *p* = 0.006) such as lymphopenia (1.15 times; *p* < 0.001), hemolytic anemia (1.9 times; *p* = 0.001), or anemia of any cause (1.29 times; *p* < 0.001), and peripheral nervous system involvement (1.87 times; *p* = 0.037) more frequently in LN. On the other hand, we documented mucocutaneous signs (1.17 fold; *p* < 0.001) such as lupus malar rash (1.21 fold; *p* = 0.016) or other skin changes (1.14 fold; *p* = 0.007), photosensitivity (1.43 fold; *p* < 0.001), and Raynaud’s phenomenon (1.56 times; *p* < 0.001) less frequently in LN patients.

### 3.3. Arterial Hypertension and Hypercholesterolemia Were the Only Internal Disease Comorbidities with a Higher Prevalence in the Lupus Nephritis Group

In general, the SLE subtype differed regarding the analyzed internal medicine comorbidities, except for arterial hypertension and hypercholesterolemia (Table 4), which were 1.75 times and 1.81 times more frequent in LN, respectively (*p* < 0.001 for both). Overall, ESKD was reported in 23 cases (6.95%) in the LN group and 3 cases (0.51%) in the non-LN group (*p* < 0.001), where it was related to concomitant internal diseases.

### 3.4. The Mortality Rates Were Similar in Lupus and Non-Lupus Nephritis Cases

Throughout the median follow-up period of 14 years, a total of 47 (5.57%) SLE patients died, with 16 (5.28%) in the LN group and 31 (5.73%) in the non-LN group (*p* = 0.79). Among the deceased, the predominant causes of death included infections (10 cases, 21.28% overall), followed by SLE exacerbation (4 cases, 8.51% overall) and malignancies (4 cases, 8.51% overall), with no significant differences observed between subgroups (*p* > 0.05 for all).

Statistically significant factors influencing mortality in all SLE patients include male sex, presence of aCL antibodies in IgG or IgM classes, presence of aβ2GPI in IgM class antibodies, internal comorbidities (arterial hypertension, diabetes mellitus, heart failure, hypercholesterolemia, atrial fibrillation, and peripheral artery disease), malignant tumor, monoclonal gammopathy of undetermined significance, thromboembolic episodes (myocardial infarction, deep vein thrombosis), rituximab administration, and certain SLE manifestations (fever, weight loss, fatigue/weakness, arthritis, pericardial or pleural effusion, hemolytic anemia, thrombocytopenia, macrophage activation syndrome, erythrocyturia or urinary casts in urine sediment, diffuse alveolar hemorrhage, and pulmonary hypertension).

### 3.5. Lupus Nephritis Was Associated with a Higher Frequency of Anti-dsDNA, Anti-Nucleosome, and Anti-Histone Antibodies

As expected, anti-dsDNA antibodies were more common in the LN group (84.44% vs. 62.48%; *p* < 0.001). Amongst the whole cohort, patients with an anti-dsDNA titer of 1:80 or more in indirect immunofluorescence had a 1.76 OR (95% CI: 1.52–2.04; *p* < 0.001) of suffering from LN. In LN, we also documented anti-nucleosome (45.89% vs. 28.62%; *p* < 0.001) and anti-histone antibodies (37.66% vs. 22.1%; *p* < 0.001) more frequently. Detailed information is shown in Table 5. Interestingly, in the presence of any of those three antibodies (*n* = 553, 72.1%), juvenile-onset SLE, recurrent fever, concomitant antiphospholipid antibodies, pleural effusion, lymphopenia, hemolytic anemia, proteinuria, leucocyturia, erythrocyturia, and granular casts in the urine sediment were reported more frequently (*p* < 0.05 for all).

Furthermore, as expected, the presence of anti-dsDNA antibodies in the whole cohort was associated with a higher number of renal exacerbations, as well as with an increased mortality rate (*p* = 0.043), atrial fibrillation (*p* = 0.011), malignancy (*p* = 0.039), and other SLE manifestations (myalgia, vasculitis, photosensitivity, and Raynaud’s phenomenon; *p* < 0.05 for all). On the other hand, anti-nucleosome antibodies in LN were associated with myocardial infarct (*p* = 0.013), as well as lymphadenopathy, arthritis, pericardial effusion, leucopenia, and central system nervous involvement (*p* < 0.05 for all). In turn, anti-histone antibodies were linked to oral and/or nasal ulcers and arthritis, arthralgia, pericardial effusion, leucopenia, and central system nervous involvement (*p* < 0.05 for all). Surprisingly, neither SLE subgroup differed in the frequency of anti-Sm antibody presence. On the other hand, anti-SSA and anti-SSB antibodies were observed more often in non-LN than in LN patients (65.94% vs. 49.37% for anti-SSA antibodies, *p* < 0.001; and 32.79% vs. 22.78% for anti-SSB antibodies, *p* = 0.002). No differences were observed in ABO blood groups and Rh blood types between both LN groups.

### 3.6. Antiphospholipid Antibodies and Arterial Thrombotic Episodes Were Reported More Frequently in the Non-Lupus Nephritis Group

Regarding antiphospholipid antibodies, there was a greater prevalence of anti-CL antibodies in the IgM class among non-LN individuals compared to those with LN (42.12% vs. 34.08%; *p* = 0.039). Furthermore, our findings indicate a 1.24-fold increase (95% CI: 1.03–1.50; *p* = 0.021) in the OR of positive aCL antibodies in the IgM class among non-LN patients as opposed to those with LN. Additionally, LN exhibited a lower incidence of arterial thrombotic episodes compared to non-LN cases (30.21% vs. 37.12%; *p* = 0.041). Notably, in LN patients, we observed a 0.85-fold decrease (95% CI: 0.74–0.98; *p* = 0.026) in the OR of arterial thrombotic episodes, with no significant difference in venous thrombotic episodes between the two groups. In contrast, individuals with the presence of aCL in the IgM class showed a higher OR for strokes (1.96-fold; 95% CI: 1.03–3.76; *p* = 0.032) and DVT (1.79-fold; 95% CI: 1.09–2.95; *p* = 0.016) within the non-LN group. For more details, see Table 4 and Table 5. We found no differences in antithrombin and protein C activity, free protein S level, level and activity of factor VIII, frequency of factor V Leiden and G20210A prothrombin gene variants between LN and non-LN patients.

### 3.7. Lupus Nephritis Is Related to a More Aggressive Immunosuppressive Treatment

The administration of immunosuppressive therapy in SLE patients is detailed in Table 6. In both SLE subgroups, corticosteroids were the most commonly used (99.39% of LN patients and 94.06% of non-LN patients). Additionally, in LN patients, chloroquine or hydroxychloroquine (99.39%), mycophenolate mofetil (65.22%), and cyclophosphamide (64.51%) were more commonly used, whereas in non-LN chloroquine or hydroxychloroquine (82.88%), azathioprine (33.45%) and methotrexate (22.54%). Obviously, more aggressive treatment modes, including mycophenolate mofetil, cyclophosphamide, rituximab, immunoglobulins, or plasmapheresis, have been reported in LN than in non-LN individuals.

### 3.8. Cluster Analysis

Next, we performed cluster analysis in both studied SLE subgroups (Table 7 and Table 8). In LN (Table 7), we revealed three different clusters: cluster 1 (*n* = 23) comprised patients with ESKD (LN patients with ESKD), cluster 2 (*n* = 203) consisted of patients without ESKD and with a time of less than one year from the first SLE symptoms to the SLE diagnosis (LN patients with early-onset SLE without ESKD), and cluster 3 (*n* = 104) consisted of patients without ESKD but with a time of at least one year from the first SLE symptoms to the SLE diagnosis (LN patients with late-onset SLE without ESKD). Compared with the remaining ones, cluster 1 was characterized by a higher frequency of pleural effusion, skin changes diagnosed as erythema, livedo racemosa, livedo reticularis, a higher frequency of class VI glomerulonephritis according to the ISN/RPS classification system in renal biopsy, and a higher rate of mortality. Patients in cluster 1 were also administered immunoglobulins and plasmapheresis more often. Moreover, cases in cluster 1 vs. cluster 2 more often had hemolytic anemia and thrombocytopenia, while women more frequently had miscarriages. Interestingly, patients in cluster 3 vs. clusters 1 and 2 were younger at the time of SLE diagnosis and suffered from arthritis more often. Clusters were comparable according to age and other internal comorbidities, autoantibody profile, and thrombotic episodes.

In the non-LN group (Table 8), we indicated two different clusters based on the time delay from the first SLE symptoms to the diagnosis. Cluster 4 had patients with less than one year from the first symptoms to diagnosis (non-LN patients with early-onset SLE) and cluster 5 had patients with one year or more (non-LN patients with late-onset SLE). The first one included 288 patients, whereas the second had 290 cases. Patients in cluster 4 were older at the time of SLE diagnosis and presented with a longer duration of the disease. Regarding clinics, both clusters were similar, except for a higher frequency of malar rash and a lower frequency of hemolytic anemia documented in those from cluster 5. They were also administered azathioprine more often.

### 3.9. Multiple Lupus Nephritis Exacerbations Are Related to the Distinct Clinical Picture

In the entire LN group, we documented renal flares in 191 (57.7%) patients, with one renal exacerbation in 58 (17.52%) cases, two renal exacerbations in 44 (14.06%) patients, and at least three renal exacerbations in 19 (5.74%) cases. The exact number of renal flares was unknown in 19 LN patients (5.74%).

Notably, LN patients with at least three renal flares exhibited distinct clinical characteristics. They were more frequently women (92.06% vs. 80.32%; *p* = 0.026), 5 years younger at the onset of SLE (medians: 25 vs. 30 years; *p* = 0.015), and 12 years younger at LN diagnosis (medians: 27 vs. 39 years; *p* < 0.001). Surprisingly, however, in those with multiple kidney exacerbations, LN was diagnosed less frequently during SLE onset (49.21% vs. 65.46%; *p* = 0.02). Furthermore, these patients reported more frequent myalgia (53.23% vs. 34.44%; *p* = 0.006), arthritis (72.13% vs. 56.22%; *p* = 0.021), nephrotic proteinuria (85.25% vs. 52.24%; *p* < 0.001), pathological urinary casts in the urine sediment (78.43% vs. 56.55%; *p* = 0.001), and end-stage kidney disease (ESKD) (14.52% vs. 5.22%; *p* = 0.006) in their medical history. Conversely, lymphopenia was the only manifestation found less frequently in those LN patients (6.67% vs. 21.1%; *p* = 0.01).

Additionally, the LN groups were similar in other SLE manifestations (*p* > 0.05 for all). Treatment modalities more frequently used in LN patients with at least three renal flares included azathioprine (69.84% vs. 46.75%; *p* = 0.001), cyclosporine A (25.4% vs. 7.76%; *p* < 0.001), mycophenolate mofetil (82.54% vs. 62.04%; *p* = 0.003), cyclophosphamide (95.24% vs. 57.32%; *p* < 0.001), and rituximab (17.74% vs. 4.08%; *p* < 0.001); however, there were no differences between groups in treatment with corticosteroids, chloroquine or hydroxychloroquine, methotrexate, belimumab, immunoglobulins, sulfasalazine, anifrolumab, plasmapheresis, and splenectomy (*p* > 0.05 for all). Additionally, both groups differed in autoantibody profile and kidney biopsy results. Those with multiple renal flares exhibited a higher frequency of anti-nucleosome (58.06% vs. 42.02%; *p* = 0.023) and anti-dsDNA antibodies (93.33% vs. 81.59%; *p* = 0.022), were class VI more frequently (6.38% vs. 1.55%; *p* = 0.012), and were class II less frequently (21.71% vs. 6.38%; *p* = 0.004) in histological investigations. In other ANA types identified by an immunoblot assay test and a histological renal biopsy, patterns were similar (*p* > 0.05 for all).

## 4. Discussion

In this study, we provide significant insights into the demographic, clinical, and laboratory profiles within a cohort comprising both LN and non-LN patients. Our findings revealed significant differences between LN and non-LN patients in several clinically relevant features. These distinctions could serve as valuable prognostic indicators for predicting which SLE patients might be at an increased risk of developing LN in the future. LN patients were younger at the time of SLE diagnosis. Obviously, women constituted the majority of cases in both SLE groups, but the percentage of men in the LN group was slightly higher. In LN, we also documented concomitant mucocutaneous manifestations, joint involvement, serositis, hematological abnormalities, and neurological involvement more frequently, along with hypertension and hypercholesterolemia as concomitant internal diseases. Patients with LN had a higher prevalence of anti-dsDNA, anti-histone, and anti-nucleosome antibodies. Conversely, aCL and anti-β2GPI in both IgM classes and thrombotic episodes (strokes and deep venous thrombosis) were reported more frequently in the non-LN group, similarly to the presence of anti-SSA and anti-SSB antibodies. In both SLE subgroups, corticosteroids were the most common therapy regimen, although as expected, LN patients were more frequently treated with immunosuppressants such as azathioprine, mycophenolate mofetil, cyclophosphamide, and rituximab.

In general, renal involvement appeared in about one-third of our SLE cohort. This frequency is similar to another report in a prospective multi-ethnic/racial SLE inception cohort, where LN occurred in 38.3% of SLE patients [14]. Furthermore, Jourde-Chiche et al. [27] highlighted the risk factors for LN relapses, encompassing antiphospholipid syndrome, higher baseline proteinuria, low C3 complement, higher Systemic Lupus Erythematosus Diseases Activity Index (SLEDAI) at inclusion, lower eGFR, lower serum albumin, lower hemoglobin levels, and lower leucocyte, lymphocyte, and eosinophil counts. In addition, Rovin et al. [15] have suggested that a decrease in complement levels and an elevation in anti-dsDNA antibodies are associated with a high likelihood of subsequent clinical LN relapse. Our findings are only partially consistent with theirs, because in addition to proteinuria, urinary protein excretion of more than 3.5 g/day, and a presence of anti-dsDNA antibodies, other significant risk factors for LN flares included female sex, younger age at LN or SLE onset, LN occurring later than SLE onset, the presence of anti-nucleosome antibodies, and several SLE manifestations such as myalgia, arthritis, and pathological urinary casts in the urine sediment. On the contrary, in our cohort, we observed that lymphopenia was associated with a lower number of renal flares. Next, we noticed a higher presence of juvenile-onset SLE in the LN group, which also stays in line with the report by Font et al. [28]. Furthermore, the shortened delay between symptom onset and SLE diagnosis in LN further underscores the urgency of promptly identifying and addressing renal involvement. Another reported risk factor for the development of LN is male sex [29], which is consistent with our findings.

The LN group was characterized by more severe clinical manifestations in our study. These patients often had general symptoms, including fever and fatigue/weakness, but also had life-threatening complications more frequently, such as serositis with pleural and pericardial effusion. Furthermore, these patients also had diffuse alveolar hemorrhage and central nervous system involvement more frequently, which interestingly were associated with specific antibody types such as anti-nucleosome or anti-histone. Neuropsychiatric SLE is a serious SLE complication [30,31]; thus, potential factors are needed to predict its development. For example, a study by Su et al. [32] specified that positive anti-SSA antibodies were related to peripheral neuropathy among LN patients and suggested their usefulness as a biomarker of this disease. We did not find this association, which may have a genetic or racial relationship. Next, the LN group had hematological manifestations such as lymphopenia, anemia, and hemolytic anemia more often. That observation also did not mirror those published by Hanly et al. [14] in a study with a large SLE cohort. Regardless of some discretions, the conditions listed above in our patients led to a worse clinical prognosis [33].

On the contrary, non-LN patients were characterized by a higher presence of mucocutaneous manifestations, photosensitivity, and Raynaud’s phenomenon with a higher prevalence of joint involvement. These observations are in line with the current literature [14]. Nevertheless, the symptoms listed above were also perceived as common disease flares in ESKD SLE patients [34], similar to Raynaud’s phenomenon. These may be a strong predictor for a poor long-term outcome in LN patients, according to a report published by Yadav et al. [35], and may therefore be linked to a worse clinical prognosis of SLE.

In turn, we did not observe any differences regarding the occurrence of several autoimmune diseases in the family history of LN and non-LN patients, but psoriasis, rheumatoid arthritis, and SLE were diagnosed most frequently in LN patients. This observation is novel, since it has previously been shown that only SLE presence in family members was a risk factor for autoimmune disorders [36].

In our dataset, we observed a higher prevalence of arterial hypertension and hypercholesterolemia in LN, which were associated with an increased mortality rate and ESKD in LN [37]; however, we found no significant differences in diabetes mellitus, heart failure, atrial fibrillation, malignant tumors, peripheral artery disease, myocardial infarct, ischemic stroke, and venous thromboembolism between the analyzed LN and non-LN patients. Thus, one might speculate that the presence of SLE itself, regardless of renal involvement, is a risk factor for those comorbidities [38,39,40,41].

The standardized mortality ratios in SLE cohorts are up to 5.3 times higher than those in age-matched healthy controls [42]. The mortality rate in our study remains comparable between LN and non-LN groups. Infections, SLE exacerbations, and emerging malignancies were the primary cause of death, consistent with the findings presented by Kandane-Rathnayake et al. [43]. These comparable mortality rates underscore the persistent need for effective management of SLE flares but also the prevention of infections whenever possible (utilizing antibiotic therapy when necessary and vaccinations) and regular oncological screenings independent from kidney involvement. Next, based on the literature, some prognostic factors are associated with higher mortality rates in SLE. They include male sex, age of at least 50 at SLE diagnosis, renal and lung involvement, thrombocytopenia, SLEDAI of at least 20 points, hypertension, ischemic heart disease, antiphospholipid syndrome (APS), and thrombotic episodes [44,45,46]. Our results align with those presented by other authors; however, other comorbidities such as diabetes mellitus, heart failure, hypercholesterolemia, atrial fibrillation, and malignancy were also associated with a higher mortality rate. Additionally, thrombocytopenia as a hematological manifestation was linked to a poor prognosis in our study, as well as hemolytic anemia of any case, including macrophage activation syndrome. What is noteworthy is that, apart from renal and lung involvements, arthritis, serositis, and general symptoms were also associated with a higher risk of death. Our data suggest that patients with SLE should undergo regular monitoring and, if necessary, immunosuppressive treatment to prevent the occurrence of serious SLE manifestations. Furthermore, an interdisciplinary approach and oncological screenings are essential in managing this group of patients.

As anticipated, LN patients exhibited a distinct pattern of autoantibody types with anti-nucleosome, anti-histone, and anti-dsDNA antibodies as the most important. Based on the literature, their association with LN is still not fully elucidated, however [47,48]. Interestingly, a study by Choi et al. [49] revealed that patients with simultaneous positivity in all of the above antibodies had higher disease activity with more advanced histopathological changes in renal biopsies, as well as a more rapid decline in renal function. We did not observe that association; however, the IV class according to ISN/RPS was the most prevalent among the 83 patients who exhibited them all, which was identified in 29 (34.94%) cases. Furthermore, anti-dsDNA antibodies were not only a predictor of LN development [29] but also poor prognosis in LN [50]. This is consistent with our results, since the presence of anti-dsDNA antibodies was linked to an increased LN exacerbation rate, malignancy, and a higher death risk. Additionally, we noted an increased incidence of anti-nucleosome antibodies in patients who experienced a myocardial infarct, which is a new finding. Interestingly, in contrast to many studies, we did not observe a higher prevalence of anti-Sm antibodies in LN cases. This may be related to the race specification, since anti-Sm antibodies are more frequently documented in African Americans, at least in some reports [51,52]. On the other hand, we observed a higher presence of rheumatoid factor and anti-SSA with anti-SSB antibodies in the non-LN group, suggesting a decreased association with LN.

The subsequent important finding of our study is the association between aCL antibodies and thrombotic episodes in non-LN subjects. The existing literature indicates that the presence of any type of antiphospholipid antibodies (aPLAs) in LN is linked to an unfavorable long-term prognosis and reduced renal survival attributed to thrombotic events [53]; however, it is necessary to note that SLE itself also increases the risk of arterial thromboembolism [54]. Moreover, according to a recent meta-analysis conducted by Domingues et al. [55], the presence of antiphospholipid antibodies in SLE is associated with a three- to five-fold increased risk of specific microvascular renal lesions; however, data from the previous literature did not report an association between antiphospholipid antibodies and LN [56,57], similar to us. All thromboembolic events occurred with a comparable incidence in both SLE groups, except for those with non-LN and the presence of aCL in the IgM class, as compared to the remaining in the same subgroup. This observation is unexpected, since the presence of LN is a strong predictor of thrombotic events, especially venous ones [58].

The detailed data on immunosuppressive therapy showed distinct patterns in medication usage between LN and non-LN cases. More than 99% of LN patients were on corticosteroids orally/intravenously, which are a flagship example of drugs used in SLE patients with affected kidneys [59]. Next, more aggressive treatment modalities, including azathioprine, cyclosporine, mycophenolate mofetil, cyclophosphamide, rituximab, immunoglobulins, and plasmapheresis were more commonly used in LN. Nevertheless, the efficacy of immunosuppressive agent-induction therapy for lupus nephritis is still being investigated [60].

Importantly, given the complexity of SLE, it is worth pointing out the role of genetic and environmental factors in the development and progression of LN. Genetic predispositions, such as specific HLA alleles and polymorphisms in immune-related genes, can increase the susceptibility to LN [61]. For example, genetic variants in genes expressed in the kidney (including *TNFRSF1B*, *KLK1*, *KLK3*, *ACE*, *AGT*, and *APOL1*) may result in increased susceptibility to kidney injury and, as a result, in progression to lupus nephritis [62]. Environmental factors, including infections, medications, and exposure to UV light, can trigger disease onset and exacerbate flares in genetically predisposed SLE/LN individuals [63,64,65]. Understanding the interplay between these genetic and environmental factors can help identify at-risk patients and develop personalized treatment strategies.

The final issue that is worth discussing is the cluster analysis based on the presence of ESKD in LN patients and the time delay from the first symptoms to SLE diagnosis in non-LN patients, which showed intriguing subgroup analyses in both LN and non-LN patients, delineating variations in clinical presentation, outcomes, and treatment responses. The groups are heterogeneous, but there are specific patterns in their clinical characteristics. Therefore, it may be possible to anticipate the course of the disease based on given features, i.e., the occurrence of certain additional complications; thus, it may help in optimizing therapy accordingly.

Our study has some limitations. Firstly, the study’s retrospective nature may introduce inherent biases in data collection and patient selection. Next, the study has a single-center design that may limit the generalizability of the results to a larger population. We did not collect patient-reported outcomes, such as quality-of-life questionnaires, which might best assess the patient’s well-being, including the impact of disease and treatment mode. Also, we did not analyze other imaging and laboratory test results, such as echocardiography. Finally, some of the presented relationships may be incidental and not represent a cause-and-effect relationship. Therefore, while our study provides valuable insights, these limitations highlight the need for cautious interpretation of the results.

## 5. Conclusions

In conclusion, the present study significantly contributes to the understanding of SLE and LN by revealing distinct demographic, clinical and laboratory features in affected individuals. Indeed, LN patients were younger at first symptoms and at disease onset but were also more often characterized by the presence of mucocutaneous, joint, and hematological manifestations and suffered more often from internal comorbidities such as hypertension, hypercholesterolemia, and end-stage kidney disease. Next, this group presented a more frequent occurrence of autoantibodies with a higher usage of immunosuppressive treatment. Furthermore, ESKD patients were characterized by a less frequent juvenile onset and a higher prevalence of skin changes and hematologic disturbances such as hemolytic anemia and thrombocytopenia.

Early identification and tailored treatment of LN are crucial given their association with more severe SLE manifestations and specific autoantibody profiles. Clinicians should prioritize monitoring high-risk patients, particularly those in the abovementioned groups, in terms of clinical and laboratory state, including autoantibody profile. Implementing comprehensive patient monitoring practices also addressing internist comorbidities, such as hypertension and hypercholesterolemia, can improve outcomes. Nevertheless, more prospective studies with diverse cohorts would be beneficial in understanding the diversity of LN and its progression in SLE patients.

## Figures and Tables

**Table 1:** Demographic characteristics of 921 patients with systemic lupus erythematosus.

Characteristics	LN Patients *n* = 331	Non-LN Patients *n* = 590	*p*-Value
**Age of onset**			
Adult onset (age of onset ≥ 18 years), *n* (%)	286 (86.9%)	544 (93.8%)	<0.001 *
Juvenile onset (age of onset < 18 years), *n* (%)	43 (13.1%)	36 (6.2%)	
**Sex of patients**			
Female, *n* (%)	276 (83.38%)	543 (92.04%)	<0.001 *
Male, *n* (%)	55 (16.62%)	47 (7.96%)	
**Disease characteristics**			
Age at first symptoms, years	28 (20.75–39)	34 (24–46)	<0.001 *
Age at onset, years	29 (22–41)	37 (27–49)	<0.001 *
Time delay between onset of symptoms and diagnosis, years	0 (0–1)	0.5 (0–3)	<0.001 *
Age at last visit, years	44 (35–57)	52 (41–63)	<0.001 *
Disease duration, years	13 (6–20)	14 (8–22)	0.76

Categorical variables are presented as numbers with percentages, continuous variables are presented as median with Q1–Q3 ranges, and an asterisk marks the statistically significant differences. Abbreviations: LN—lupus nephritis, *n*—number.

**Table 2:** Characteristics of treatment in 180 lupus nephritis patients who underwent kidney biopsy.

Treatment	I *n* = 3	II *n* = 33	III *n* = 26	IV *n* = 91	V *n* = 22	VI *n* = 5	*p*-Value
Glucocorticoids oral and/or intravenous, *n* (%)	3 (100.0%)	33 (100.0%)	25 (96.2%)	90 (98.9%)	22 (100.0%)	5 (100.0%)	0.31
Chloroquine or hydroxychloroquine, *n* (%)	2 (66.7%)	21 (63.6%)	18 (69.2%)	56 (61.5%)	12 (54.5%)	4 (80.0%)	0.79
Azathioprine, *n* (%)	2 (66.7%)	16 (48.5%)	14 (53.8%)	43 (47.3%)	12 (54.5%)	4 (80.0%)	0.71
Methotrexate, *n* (%)	1 (33.3%)	2 (6.1%)	5 (19.2%)	14 (15.4%)	6 (27.3%)	2 (40.0%)	0.24
Cyclosporine, *n* (%)	0 (0.0%)	2 (6.1%)	2 (7.7%)	16 (17.6%)	5 (22.7%)	1 (20.0%)	0.42
Belimumab, *n* (%)	1 (33.3%)	1 (3.0%)	1 (3.8%)	8 (8.8%)	0 (0.0%)	0 (0.0%)	0.21
Mycophenolate mofetil, *n* (%)	1 (33.3%)	20 (60.6%)	15 (57.7%)	72 (79.1%)	18 (81.8%)	5 (100.0%)	0.06
Cyclophosphamide, *n* (%)	1 (33.3%)	15 (45.5%)	18 (69.2%)	77 (84.6%)	18 (81.8%)	5 (100.0%)	<0.001 *
Rituximab, *n* (%)	0 (0.0%)	1 (3.0%)	0 (0.0%)	10 (11.0%)	3 (13.6%)	0 (0.0%)	0.30
Immunoglobulins, *n* (%)	0 (0.0%)	1 (3.0%)	1 (3.8%)	2 (2.2%)	2 (9.1%)	1 (20.0%)	0.34
Plasmapheresis, *n* (%)	0 (0.0%)	2 (6.1%)	1 (3.8%)	3 (3.3%)	2 (9.1%)	2 (40.0%)	0.027 *
Sulfasalazine, *n* (%)	1 (33.3%)	0 (0.0%)	0 (0.0%)	4 (4.4%)	2 (9.1%)	0 (0.0%)	0.053

Categorical variables are presented as numbers and an asterisk marks the statistically significant differences. Abbreviations: *n*—number.

**Table 3:** Cumulative frequencies of systemic involvement in all enrolled patients.

Clinical Manifestations	LN Patients *n* = 331	Non-LN Patients *n* = 590	*p*-Value
**Constitutional manifestations, *n* (%)**	268 (80.97%)	461 (78.14%)	0.35
Fever, *n* (%)	166 (52.87%)	233 (39.9%)	<0.001 *
Fatigue/weakness, *n* (%)	222 (70.03%)	366 (62.89%)	0.037 *
Myalgias, *n* (%)	122 (38.49%)	222 (38.21%)	0.99
Weight loss, *n* (%)	81 (25.80%)	121 (20.75%)	0.10
Lymphadenopathy, *n* (%)	64 (20.25%)	114 (19.52%)	0.86
**Mucocutaneus manifestations, *n* (%)**	248 (74.92%)	515 (87.30%)	<0.001 *
Lupus malar rash, *n* (%)	130 (39.88%)	285 (48.39%)	0.016 *
Discoid rash, *n* (%)	22 (6.77%)	52 (8.83%)	0.33
Urticaria, *n* (%)	26 (8.00%)	51 (8.66%)	0.83
Cutaneous vasculitis, *n* (%)	23 (7.08%)	36 (6.11%)	0.67
Alopecia, *n* (%)	85 (26.07%)	161 (27.33%)	0.74
Oral and/or nasal ulcers, *n* (%)	51 (15.69%)	92 (15.62%)	0.95
Photosensitivity, *n* (%)	89 (27.38%)	231 (39.22%)	<0.001 *
Other skin changes ^1^, *n* (%)	206 (62.61%)	421 (71.48%)	0.007 *
**Joint manifestations, *n* (%)**	279 (84.29%)	548 (92.88%)	<0.001 *
Arthritis, *n* (%)	194 (59.69%)	385 (65.59%)	0.09
Arthralgia, *n* (%)	279 (84.80%)	545 (92.37%)	<0.001 *
**Serositis, *n* (%)**	122 (37.08%)	112 (18.98%)	<0.001 *
Pleural effusion, *n* (%)	90 (27.44%)	73 (12.37%)	<0.001 *
Pericardial effusion, *n* (%)	75 (23.44%)	62 (10.56%)	<0.001 *
Pericarditis, *n* (%)	12 (3.65%)	25 (4.24%)	0.79
**Hematological manifestations, *n* (%)**	315 (95.17%)	529 (89.66%)	0.006 *
Leucopenia ^2^, *n* (%)	209 (65.11%)	362 (62.31%)	0.44
Lymphopenia ^3^, *n* (%)	261 (83.65%)	414 (72.89%)	<0.001 *
Anemia ^4^, *n* (%)	272 (84.47%)	381 (65.69%)	<0.001 *
Hemolytic anemia ^5^, *n* (%)	42 (30.43%)	42 (16.03%)	0.001 *
Thrombocytopenia ^6^, *n* (%)	110 (34.16%)	182 (31.33%)	0.42
Direct Coombs test, *n* (%)	29 (36.25%)	31 (41.33%)	0.63
Macrophage activation syndrome, *n* (%)	5 (1.53%)	3 (0.51%)	0.23
Thrombotic thrombocytopenic purpura ^7^, *n* (%)	1 (0.30%)	1 (0.17%)	0.75
**Kidney involvement, *n* (%)**	331 (100%)	0 (0%)	<0.001 *
24 h urinary protein excretion > 0.5 g/day, *n* (%)	300 (96.46%)	0 (0%)	<0.001 *
24 h urinary protein excretion > 3.5 g/day, *n* (%)	158 (58.74%)	0 (0%)	<0.001 *
Urinary casts, *n* (%)	138 (61.06%)	0 (0%)	<0.001 *
Erythrocyturia, *n* (%)	229 (84.81%)	0 (0%)	<0.001 *
Leukocyturia, *n* (%)	242 (84.62%)	0 (0%)	<0.001 *
**Neurological abnormality, *n* (%)**	59 (17.82%)	45 (7.63%)	<0.001 *
Central nervous system involvement, *n* (%)	44 (13.37%)	26 (4.41%)	<0.001 *
Peripheral nervous system involvement, *n* (%)	24 (7.29%)	23 (3.90%)	0.037
**Raynaud’s phenomenon, *n* (%)**	61 (18.48%)	170 (28.81%)	<0.001 *
**Lung involvement, *n* (%)**	30 (9.06%)	49 (8.31%)	0.79
Interstitial lung disease, *n* (%)	19 (5.76%)	30 (5.08%)	0.78
Diffuse alveolar hemorrhage, *n* (%)	8 (2.42%)	3 (0.51%)	0.024 *
Pulmonary hypertension, *n* (%)	9 (2.80%)	22 (3.75%)	0.57
**Lupoid hepatitis, *n* (%)**	13 (3.94%)	31 (5.25%)	0.46

Categorical variables are presented as numbers with percentages, and an asterisk marks the statistically significant differences. Abbreviations: *n*—number; LN—lupus nephritis. ^1^—erythema, livedo racemosa, livedo reticularis; ^2^—<4000/mm^3^ or diagnosis in a medical history; ^3^—<1500/mm^3^ or diagnosis based on a medical history; ^4^—≤12 g/dL in women, ≤13.5 g/dL in men, or diagnosis based on medical history; ^5^—anemia with a positive direct Coombs test or anemia with a decreased level of haptoglobin or diagnosis based on a medical history; ^6^—<100,000/mm^3^ or diagnosis based on a medical history; ^7^—confirmed with ADAMTS-13 level.

**Table 4:** Cumulative frequencies of comorbidities in all included patients.

Comorbidities ^1^	LN Patients *n* = 331	Non-LN Patients *n* = 590	*p*-Value
Hypertension, *n* (%)	241 (72.81%)	246 (41.69%)	<0.001 *
Diabetes mellitus, *n* (%)	38 (11.48%)	58 (9.83%)	0.50
Heart failure ^2^, n (%)	23 (6.95%)	24 (4.07%)	0.08
Hypercholesterolemia ^3^, *n* (%)	223 (67.58%)	220 (37.29%)	<0.001 *
Atrial fibrillation, *n* (%)	14 (4.23%)	19 (3.22%)	0.54
Peripheral artery disease, *n* (%)	11 (3.33%)	39 (6.61%)	0.05
End-stage kidney disease, *n* (%)	23 (6.97%)	2 (0.34%)	<0.001 *
Monoclonal gammopathy of undetermined significance, *n* (%)	9 (2.72%)	10 (1.69%)	0.42
Malignant tumor, *n* (%)	31 (9.37%)	61 (10.37%)	0.71
Artery thrombotic episode, *n* (%)	100 (30.21%)	219 (37.12%)	0.041 *
Stroke, *n* (%)	21 (6.34%)	52 (8.81%)	0.23
Transient ischemic attack, *n* (%)	6 (1.81%)	10 (1.69%)	0.89
Myocardial infarct, *n* (%)	88 (26.59%)	184 (31.19%)	0.16
Thrombotic episode in another artery, *n* (%)	7 (2.11%)	15 (2.54%)	0.85
Venous thrombotic episode, *n* (%)	61 (18%)	108 (18%)	0.97
Deep venous thrombosis, *n* (%)	50 (15%)	91 (15%)	0.97
Pulmonary embolism, *n* (%)	14 (4%)	25 (4%)	0.88
Deep venous thrombosis and pulmonary embolism, *n* (%)	8 (2%)	11 (2%)	0.75
Thrombotic episode in another venous, *n* (%)	8 (2%)	11 (2%)	0.75
Miscarriage, *n* (%)	32 (13.06%) ^4^	79 (18.16%) ^4^	0.11

Categorical variables are presented as numbers with percentages, and an asterisk marks the statistically significant differences. ^1^—comorbidities present or in the past; ^2^—symptoms of heart failure or LVEF ≤ 40% or a diagnosis based on medical history; ^3^—LDL > 3 mmol/L or pharmacotherapy with statin or a diagnosis based on medical history; ^4^—% of women with miscarriage from number of women with systemic lupus erythematous. Abbreviations: LDL—low-density lipoprotein, LN—lupus nephritis, LVEF—left ventricular ejection fraction, *n*—number.

**Table 5:** Laboratory findings in all included patients.

Laboratory Parameter (Number of Patients with Analyzed Parameter)	LN Patients *n* = 331	Non-LN Patients *n* = 590	*p*-Value
Rheumatoid factor, *n* (%)	33 (20.89%)	130 (38.01%)	<0.001 *
*ANA—IIF assay, n (%)*	331 (100%)	590 (100%)	
Anti-SSA antibodies ^1^, *n* (%)	156 (49.37%)	364 (65.94%)	<0.001 *
Anti-SSB antibodies ^1^, *n* (%)	72 (22.78%)	181 (32.79%)	0.002 *
Anti-histone antibodies ^1^, *n* (%)	119 (37.66%)	122 (22.1%)	<0.001 *
Anti-nucleosome antibodies ^1^, *n* (%)	145 (45.89%)	158 (28.62%)	<0.001 *
Anti-Smith antibodies ^1^, *n* (%)	41 (13.08%)	71 (12.89%)	0.97
Anti-RNP antibodies ^1^, *n* (%)	77 (24.44%)	114 (20.69%)	0.23
Anti-dsDNA antibodies ^1^, *n* (%)	163 (51.58%)	174 (31.75%)	<0.001 *
Anti-dsDNA antibodies ^2^, *n* (%)	266 (84.44%)	323 (62.48%)	<0.001 *
Anti-PR3 antibodies ^3^, *n* (%)	5 (6.49%)	2 (2.99%)	0.56
Anti-MPO antibodies ^3^, *n* (%)	9 (11.11%)	5 (7.14%)	0.58
*Antiphospholipid antibodies*			
Lupus anticoagulant, *n* (%)	64 (25.60%)	128 (30.62%)	0.19
Anti-cardiolipin antibodies IgG or IgM, *n* (%)	150 (55.56%)	270 (56.84%)	0.83
Anti-cardiolipin antibodies IgG, *n* (%)	120 (44.78%)	187 (40.30%)	0.27
Anti-cardiolipin antibodies IgM, *n* (%)	91 (34.08%)	195 (42.12%)	0.039 *
Anti-β2 glycoprotein I IgG or IgM, *n* (%)	46 (20.91%)	110 (29.1%)	0.035 *
Anti-β2 glycoprotein I IgG, *n* (%)	29 (13.62%)	66 (17.84%)	0.22
Anti-β2 glycoprotein I IgM, *n* (%)	27 (12.68%)	80 (21.68%)	0.009 *

Categorical variables are presented as numbers with percentages, and an asterisk marks the statistically significant differences. ^1^—Immunoblotting assay; ^2^—CLIFT (the *Crithidia luciliae* immunofluorescence test); ^3^—ELISA (Enzyme-Linked Immunosorbent Assay). Abbreviations: ANA—anti-nuclear antibodies, dsDNA—double stranded DNA, IIF—indirect immunofluorescence, MPO—myeloperoxidase, PR3—proteinase 3, RNP—ribonucleoprotein, LN—lupus nephritis, *n*—number.

**Table 6:** Treatment received by all enrolled patients.

Treatment	LN Patients *n* = 331	Non-LN Patients *n* = 590	*p*-Value
Glucocorticoids oral and/or intravenous, *n* (%)	327 (99.39%)	554 (94.06%)	<0.001 *
Chloroquine or hydroxychloroquine, *n* (%)	328 (99.39%)	489 (82.88%)	0.06
Azathioprine, *n* (%)	166 (51.08%)	197 (33.45%)	<0.001 *
Methotrexate, *n* (%)	56 (17.39%)	133 (22.54%)	0.06
Cyclosporine, *n* (%)	38 (11.73%)	39 (6.62%)	0.036 *
Belimumab, *n* (%)	19 (5.92%)	21 (3.57%)	0.09
Mycophenolate mofetil, *n* (%)	210 (65.22%)	90 (15.28%)	<0.001 *
Cyclophosphamide, *n* (%)	209 (64.51%)	72 (12.22%)	<0.001 *
Rituximab, *n* (%)	21 (6.54%)	8 (1.36%)	<0.001 *
Immunoglobulins, *n* (%)	17 (5.28%)	11 (1.87%)	0.010 *
Plasmapheresis, *n* (%)	27 (8.41%)	4 (0.68%)	<0.001 *
Sulfasalazine, *n* (%)	12 (3.73%)	36 (6.11%)	0.13
Anifrolumab, *n* (%)	4 (1.24%)	6 (1.02%)	0.35
Splenectomy, *n* (%)	1 (0.31%)	4 (0.68%)	0.80

Categorical variables are presented as numbers with percentages, and an asterisk marks the statistically significant differences. Abbreviations: LN—lupus nephritis, *n*—number.

**Table 7:** Three clusters among lupus nephritis patients based on the time from the first systemic lupus erythematosus symptoms to the disease diagnosis and the presence of end stage kidney disease.

Features	Cluster 1 LN Patients with ESKD *n* = 23	Cluster 2 LN Patients with Early-Onset SLE without ESKD *n* = 203	Cluster 3 LN Patients with Late-Onset SLE without ESKD *n* = 104	*p*-Value
Juvenile onset (age of onset < 18 years), *n* (%)	17 (73.91%)	172 (84.31%) ^#^	97 (95.10%) *	0.003
Other skin changes ^1^, *n* (%)	8 (34.78%)	125 (61.88%) *	73 (70.19%) **	0.007
Pleural effusion, *n* (%)	12 (52.17%)	55 (27.23%) *	23 (22.33%) **	0.002
Arthritis, *n* (%)	10 (43.48%)	112 (56.57%) ^#^	72 (69.23%) *	0.003
Hemolytic anemia ^2^, *n* (%)	7 (70.00%)	24 (29.27%) *	11 (23.91%)	0.020
Thrombocytopenia ^3^, *n* (%)	14 (60.87%)	55 (27.92%) **^,#^	41 (40.20%)	0.002
Miscarriages, *n* (%)	5 (26.32%)	13 (8.67%) *	14 (18.18%)	0.030
LN class VI ^4^, *n* (%)	3 (27.27%)	1 (0.88%) **	1 (1.72%) *	0.020
Death, *n* (%)	6 (37.5%)	8 (4.12%) **	2 (2.15%) **	<0.001
Plasmapheresis, *n* (%)	8 (34.78%)	14 (7.18%) **	5 (4.85%) **	0.004

Categorical variables are presented as numbers with percentages. *—*p* < 0.05 in comparison with cluster 1; **—*p* < 0.01 in comparison with cluster 1; ^#^—*p* < 0.05 in comparison with cluster 3; ^1^—erythema, livedo racemosa, livedo reticularis; ^2^—anemia with a positive direct Coombs test, anemia with a decreased level of haptoglobin, or a diagnosis based on medical history; ^3^—<100,000/mm^3^ or diagnosis based on medical history; ^4^—according to the International Society of Nephrology/Renal Pathology Society criteria. In two (0.6%) LN cases, the time of first kidney manifestation in the SLE course was unknown. Abbreviations: ESKD—end-stage kidney disease, LN—lupus nephritis, *n*—number, SLE—systemic lupus erythematosus.

**Table 8:** Two clusters among non-lupus nephritis patients based on the time from the first systemic lupus erythematosus symptoms to the SLE diagnosis.

Features	Cluster 4 Non-LN Patients with Early-Onset SLE *n* = 288	Cluster 5 Non-LN Patients with Late-Onset SLE *n* = 290	*p*-Value
Juvenile onset (age of onset < 18 years), *n* (%)	259 (89.93%)	283 (97.59%)	<0.001
Lupus malar rash, *n* (%)	155 (53.82%)	126 (43.60%)	0.016
Direct Coombs test, *n* (%)	12 (29.27%)	19 (55.88%)	<0.001
Diabetes mellitus, *n* (%)	36 (12.50%)	18 (6.21%)	<0.001
Azathioprine, *n* (%)	114 (39.58%)	82 (28.28%)	0.004

Categorical variables are presented as numbers with percentages. In two (0.6%) LN cases, the time of first kidney manifestation in the SLE course was unknown. Abbreviations: LN—lupus nephritis, *n*—number, SLE—systemic lupus erythematosus.

## Data Availability

The data presented in this study are available on reasonable request from the corresponding author.
